# Mixed Nut Challenge Test (MixNut) as an Efficient Procedure in the Management of Lipid Transfer Protein Allergy

**DOI:** 10.3390/nu17243822

**Published:** 2025-12-06

**Authors:** Diana Betancor, Elisa Haroun, Manuel De las Heras Gozalo, Carlos Pastor-Vargas, Javier Cuesta-Herranz

**Affiliations:** 1 Department of Allergy, Hospital Universitario Fundación Jiménez Diaz, DíazAv Reyes Católicos, 2, 28040 Madrid, Spain; diana13_b@hotmail.com (D.B.); mheras@fjd.es (M.D.l.H.G.); j.cuestaherranz@gmail.com (J.C.-H.); 2 Department of Allergy, Hospital Universitario Infanta Leonor, 28031 Madrid, Spain; elisaharoun@hotmail.com; 3 Department of Biochemistry and Molecular Biology, Universidad Complutense de Madrid, 28040 Madrid, Spain

**Keywords:** lipid transfer protein, oral food challenge, nut allergy, mixed nut challenge

## Abstract

**Background/Objectives**: Lipid transfer protein (LTP) syndrome is a leading cause of primary food allergy in Mediterranean countries, often associated with severe reactions. Due to in vitro cross-reactivity among plant foods, clinical manifestations are unpredictable, frequently requiring multiple oral food challenges (OFC) to assess nut tolerance. These procedures increase healthcare burden and patient anxiety. This study evaluated the safety and utility of a mixed-nut oral food challenge (MixNut) in LTP-sensitized patients. **Methods**: In this prospective observational multi-center study, patients with LTP syndrome were enrolled. Group A included individuals allergic to fruits or vegetables who had avoided nuts; Group B included patients with suspected or confirmed nut allergy. Participants underwent a MixNut challenge comprising 2–4 nuts (≥3 g protein per nut). **Results**: Nineteen patients (73.7% male; median age 32.5 years) underwent MixNut, testing 52 individual nuts. All challenges were negative. The MixNut approach reduced the number of OFC by 63% (from 52 to 19) and total testing time from 208 to 76 h. Specific IgE levels to LTP allergens (Pru p 3, Cor a 8, Ara h 9) varied widely and did not predict clinical reactivity. **Conclusions**: MixNut is an efficient diagnostic tool for LTP syndrome, significantly reducing testing time, costs, and patient burden. It facilitates accurate dietary management and prevents unnecessary food avoidance. Further studies should optimize MixNut protocols and identify predictive markers for clinical reactivity.

## 1. Introduction

Lipid transfer protein (LTP) is recognized as the main cause of primary food allergy in Mediterranean countries and is frequently associated with severe allergic reactions, including anaphylaxis. As members of the LTP family, these panallergens are small, highly stable proteins that resist heat and digestion, which explains their strong allergenic potential and capacity to trigger systemic reactions [1]. Because they are widely distributed across the plant kingdom, sensitization to LTPs results in a broad, heterogeneous clinical spectrum, ranging from mild oral allergy syndrome to life-threatening anaphylaxis. This extensive distribution also leads to unpredictable cross-reactivity patterns among botanically unrelated foods. In patients with LTP syndrome, nuts, particularly walnuts, hazelnuts, and peanuts, are the second most frequent cause of anaphylaxis after Rosaceae fruits such as peach or apple [1,2].

The diagnostic approach to food allergy relies primarily on clinical history, supported by in vivo tests such as skin prick tests (SPTs) and in vitro specific Immunoglobulin E (IgE) assays. However, in many LTP-sensitized patients, these tests correlate poorly with clinical reactivity, and the oral food challenge (OFC) remains the gold standard. When several nuts are involved, multiple OFCs are often required to determine individual tolerance profiles. Although effective, this strategy carries notable drawbacks: increased healthcare costs, prolonged evaluation timelines, and heightened patient anxiety. In addition, time away from work or school during extensive testing increases the indirect burden of disease. Misdiagnosis or overly cautious management may also result in unnecessary dietary restrictions, reduced nutritional variety, and a lower quality of life.

To overcome these limitations, a mixed nut challenge (MixNut) has been proposed as a time- and cost-efficient alternative, particularly in cases where tolerance to different nuts is uncertain [3]. Existing mixed nut oral food challenge (MNOFC) protocols have been evaluated mainly in pediatric populations, especially among children with peanut allergy [4,5] or unclear tolerance to multiple nuts [3,5,6]. However, data on its use in adults are scarce, and no evidence is currently available regarding its application in patients with LTP syndrome, despite the high prevalence of this condition in Southern Europe.

The aim of the present study was to evaluate and describe our clinical experience using a standardized MixNut protocol in patients diagnosed with LTP syndrome, in order to assess its safety, efficiency, and clinical utility within routine allergy management.

## 2. Materials and Methods

### 2.1. Study Design and Setting

We conducted a prospective observational cohort study to evaluate the efficiency of a selected MixNut challenge for assessing nut tolerance in patients with LTP allergy syndrome. The study was carried out in the outpatient allergy clinics of Fundación Jiménez Díaz University Hospital and Infanta Leonor Hospital (Madrid, Spain) between 2023 and 2025. All procedures were supervised by an allergist and specialized nursing staff trained in the management of allergic reactions.

### 2.2. Participants

Two groups of sensitized patients were included:

- Group A: Individuals with IgE-mediated allergy to fruits or vegetables attributed to LTP syndrome who had avoided nuts since the onset of their condition. They underwent a MixNut challenge to determine the presence or absence of clinical reactivity.
- Group B: Individuals with a history suggestive of IgE-mediated allergy to one or several nuts and confirmed LTP sensitization. They underwent a MixNut challenge, including 2, 3, or 4 nuts selected according to patient preference and physician recommendation.

### 2.3. Exclusion Criteria

Participants were excluded if they had:

1. A prior episode of severe nut-induced anaphylaxis, or
2. Sensitization to purified seed storage proteins measurable by ImmunoCAP (Ana o 3, Ara h 1, Ara h 2, Ara h 3, Ara h 6, Jug r 1, Cor a 14), given their association with severe reactions.

### 2.4. MixNut Challenge Protocol

The protocol was based on previous mixed-nut studies [7,8] and international guidelines recommending administration of at least 3 g of protein from each nut during a challenge [9]. The procedure began with a small dose of each nut administered separately (step 1), followed by incremental combined doses every 15 min (steps 2–6). Patients were then observed for two hours after completing the challenge. A summary is provided in Table 1.

- Step 1: Administer the first dose of each nut individually, with 15-min intervals. The physician selected the order, typically starting with the nut expected to be most allergenic.
- Step 2: Administer the second dose of all nuts together; observe for 15 min.
- Steps 3–4: Administer the third and fourth combined doses, each followed by a 15-min observation period.
- Step 5: Administer the remaining target total dose.
- Step 6: If tolerated, observe for an additional 2 h to detect delayed reactions.

Total administered doses ranged from 15 to 25 g per nut, following European recommendations. Sunflower seed was an exception due to its small size; meeting standard protein thresholds would require an excessively large number of seeds, which could compromise tolerability. A reduced, adapted amount was therefore used.

### 2.5. Criteria for a Positive Challenge

The procedure was stopped if objective allergic symptoms appeared, including urticaria, angioedema, respiratory or gastrointestinal symptoms, or other systemic manifestations absent at baseline. Patients were managed according to emergency protocols, and the challenge was classified as positive.

### 2.6. Data Collection

Demographic and clinical data were recorded for all participants. Total IgE and specific IgE (sIgE) levels were quantified using the ImmunoCAP^®^ system (Phadia, Uppsala, Sweden), a validated fluorescence enzyme immunoassay. Blood samples were obtained at baseline and processed according to the manufacturer’s instructions. Results were expressed in kUA/L. The following allergens were quantified: LTP-related components (Pru p 3, Cor a 8, Ara h 9), PR-10 (Pru p 1), profilin (Pru p 4), and storage proteins (Ana o 3, Ara h 1, Ara h 2, Ara h 3). Values ≥ 0.35 kUA/L were considered positive. The performance and clinical validity of this assay have been previously documented [10].

The study was approved by the Fundación Jiménez Díaz Ethics Committee (Ref. PIC201-20_FJD).

### 2.7. Statistical Analysis

Quantitative variables were described as mean and standard deviation or median (IQR), depending on distribution. Qualitative variables were expressed as absolute and relative frequencies. Group comparisons were performed using the chi-square or Fisher’s exact test for qualitative variables and ANOVA or Kruskal–Wallis test for quantitative variables. Statistical analyses were performed using GraphPad Instat6 (GraphPad Software Inc., San Diego, CA, USA). A *p*-value < 0.05 was considered statistically significant.

## 3. Results

### 3.1. Patients’ General Characteristics

Nineteen patients underwent MixNut: 6 from group A and 13 from group B. The majority were male (14 patients, 73.7%) with a median age of 32.5 years (range 8–71 years). 78.9% of the participants had allergic rhinitis, and 31.6% had concomitant asthma. Systemic reactions were the most common clinical presentation (94.7%). None of the initial reactions required cofactors. Detailed information about the patient’s demographic and clinical characteristics is summarized in Table 2.

### 3.2. Patients’ Characteristics Divided into Group A and Group B

All MixNut yielded a negative result. SIgE levels to LTPs allergens such as Pru p 3, Cor a 8, and Ara h 9 varied widely among participants with independence of challenge result. Nuts involved in the MixNut, as well as specific IgE values, are given in Table 3.

### 3.3. Comparison Between Group A and Group B

Total IgE levels were markedly elevated in both groups, with wide inter-individual variability. Although Group B showed higher median specific IgE levels to Pru p 3 (4.8 kUA/L vs. 9.4 kUA/L), this difference was not statistically significant (*p* > 0.05). In contrast, specific IgE levels to Cor a 8 (1.07 kUA/L vs. 1.64 kUA/L) and Ara h 9 (1.38 kUA/L vs. 1.69 kUA/L) were comparable between groups.

The mean specific IgE level to walnut was significantly higher in Group B (2.58 kUA/L) than in Group A (0.42 kUA/L) (*p* = 0.02), consistent with the higher prevalence of walnut-allergic patients in Group B. No significant differences were observed for the remaining nuts. Table S1 (Supplementary Materials) summarizes specific IgE levels to LTP-related allergens and individual nuts in both groups.

Only one patient developed oral allergy syndrome (OAS), classified within Group B. This individual showed concomitant sensitization to Cor a 1 (PR-10 family), typically associated with mild, non-systemic reactions. No specific IgE value showed a statistically significant association with the presence of systemic symptoms or anaphylaxis.

### 3.4. Clinical Results of the MixNut Challenge

A total of 52 individual nuts were evaluated across 19 MixNut procedures. This indicates that, without the MixNut protocol, 52 separate conventional OFC would have been required. By using the MixNut approach, only 19 mixed-nut OFC were necessary, thereby avoiding 33 individual nut challenges, which corresponds to a 63% reduction in testing days. Based on AAAAI-EAACI guideline recommendations8, which estimate a minimum duration of 4 h per OFC, the implementation of MixNut significantly reduces the cumulative testing time: from 208 h with standard OFC to 76 h (a reduction of 63.5% hours).

## 4. Discussion

This prospective study, conducted in two tertiary centers between 2023 and 2025, evaluated the safety and usefulness of the MixNut challenge in adults with LTP syndrome. Participants were selected according to predefined criteria to balance safety with diagnostic accuracy. The protocol, aligned with international recommendations, enabled consistent dose escalation and systematic monitoring. The structured design strengthens the evaluation of MixNut as a diagnostic tool capable of reducing the number of individual OFCs, shortening assessment time, and decreasing patient burden.

Our findings indicate that MixNut is an efficient method for managing LTP-sensitized individuals. Assessing several nuts within one protocol reduced the number of tests by 63.5%, yielding substantial time and resource savings for both patients and healthcare institutions. MixNut also facilitated accurate dietary recommendations, reducing unnecessary restrictions. Patients in Group A—those who had avoided nuts since developing LTP-related symptoms—were able to safely reintroduce them. Similarly, patients in Group B, despite confirmed allergy to one nut, often tolerated others, underscoring that sensitization alone does not predict clinical reactivity. These observations support previous evidence showing that food-specific IgE values, particularly <5 kUA/L, are weak predictors of clinical allergy in the absence of compatible symptoms.

There is currently no standardized MixNut protocol. Reported approaches vary widely, with administered doses ranging from 5 to 17 g per nut. Our protocol followed guideline recommendations, providing at least 3 g of protein per nut (15–25 g of nut weight), exceeding the doses used in previous studies. Nut selection was based on clinical probability and patient preference, typically involving two to four nuts per challenge, consistent with earlier publications.

Most research on mixed-nut challenges has been conducted in children, with only one study including adolescents. This is the first investigation focused primarily on adults, demonstrating that MixNut is safe and effective beyond the pediatric population. Reported positive challenge rates in previous studies range from 0% to 33%, influenced by differences in sensitization patterns, protocols, and nut selection. In our cohort, no patient had a positive MixNut. This likely reflects careful selection of both patients and test nuts, contributing to the absence of adverse reactions and supporting the value of individualized decision-making. These results align with prior Mediterranean studies using selected nuts, although no direct comparison is yet possible because this is the first adult LTP-focused MixNut study.

Some protocols incorporate nut mixtures prepared at home. This approach limits the ability to identify the causative nut in case of a reaction, and dose accuracy is compromised by uneven mixing and variable ingestion. Homemade preparations also increase the risk of cross-contamination and inconsistent nut proportions. In contrast, incremental administration of individual nuts, as implemented in our protocol, provides better control of exposure and improves reproducibility. Although nut selection in our study was individualized based on physician assessment and patient preference (introducing some variability), this reflects real clinical practice and enhances patient adherence.

This study has several strengths. It was conducted prospectively across two centers, improving reliability and generalizability. The MixNut protocol was clearly defined and guideline-based. Adult patients with LTP syndrome were specifically included, addressing a gap in existing literature. Both safety and efficiency were systematically evaluated, and sIgE measurements were obtained using a validated assay. Rigorous inclusion and exclusion criteria helped minimize confounding effects from severe nut allergy or storage protein sensitization.

However, the sample size was relatively small, and only patients without prior severe nut-induced anaphylaxis were included, which may limit representativeness. Additionally, as none of the LTP-related reactions required cofactors, challenges were performed under stable conditions. Reproducing cofactor-dependent reactions in a controlled setting is inherently difficult, and previous studies have suggested cofactors are involved in fewer than 10% of LTP-related reactions. Given the sample size, cofactor-dependent responses may not have been captured.

Mixed-nut challenges represent an efficient strategy for evaluating tolerance to multiple nuts within a single session, optimizing both clinical time and resource allocation. This approach is particularly advantageous in low-risk patients, in whom the likelihood of significant reactions is low. By consolidating evaluation into one procedure, patient anxiety is reduced, and workflow is improved. The main limitation is that a positive reaction necessitates subsequent individual OFCs to identify the specific culprit nut. Nevertheless, MixNut remains a valuable initial tool to rule out broad nut allergy and streamline diagnostic pathways.

## 5. Conclusions

In conclusion, our study supports the use of MixNut as a reliable method to assess nut tolerance in patients with LTP syndrome. By allowing the simultaneous assessment of multiple nuts, it significantly reduces the number of conventional oral food challenges, decreases testing time, and minimizes patient burden, while supporting accurate dietary management and preventing unnecessary food avoidance. Although further studies with larger and more diverse populations are needed to confirm these findings and identify predictive biomarkers of clinical reactivity, MixNut represents a promising approach to streamline allergy evaluation in LTP-sensitized patients.

## Figures and Tables

**Table 1:** MixNut oral challenge protocol.

	Almond/ Hazelnut/Cashew	Peanut/Pistachio	Walnut	Sunflower Seed
Weight of the nut *	1.2–1.5 g per unit	0.7 g per unit	2.5 g per halves	0.05 g per unit
1st	1/4	1/2	1/4	1
2nd	1	2	1	5
3rd	3	6	2	10
4th	6	8	3	20
5th	Rest	Rest	Rest	Rest
Cumulative dose	12 units (14.5–18 g of nuts)	20 units (14 g of nuts)	10 halves (25 g of walnuts)	70 units (3.5 g of sunflower seed)

* Weight of each nut is approximately measured in grams; the weight has been established by the Agricultural Research Service, FoodData Central, available at https://fdc.nal.usda.gov. The dose of nut protein given to the patient has been established at 3 g, based on the studies of Sampson HA et al. [9].

**Table 2:** Patient’s demographic and clinical characteristics.

	Patients Number	Age	Gender	Rhinitis	Asthma	Diagnosed Allergy Due to LTP	Allergic Clinic for the First Allergic Reaction
**Group A**	1	8	F	Y	N	Rosacea fruit (C + Ig + OFC)	Sys
	2	34	F	Y	Y	Rosacea fruit (C + Ig)	Sys
	3	50	M	N	Y	Vegetables (C + SPT)	Sys
	4	45	F	N	N	Rosacea fruit (C + Ig)	An
	5	36	F	Y	Y	Rosacea (C + Ig)	Sys
	6	71	M	Y	N	Pt (C + SPT + Ig + OFC)	An
**Group B**	7	10	M	Y	Y	Hz (C + SPT + Ig + OFC)	Sys
	8	19	M	Y	Y	Wn (C + Ig)	OAS
	9	30	F	Y	N	S (C + Ig)	Sys
	10	17	M	Y	N	Hz (C + Ig + OFC)	Sys
	11	35	M	Y	N	Wn (C + SPT + Ig)	An
	12	40	M	N	N	Pt (C + Ig)	Sys
	13	17	M	Y	N	Mixed nuts (C + SPT)	Sys
	14	19	M	N	N	Mixed nuts (C + SPT)	An
	15	11	M	Y	Y	Mixed nuts (C + SPT)	Sys
	16	41	M	Y	N	Mixed nuts (C + SPT)	Sys
	17	71	M	Y	N	Pt (C + OFC + SPT)	Sys
	18	24	M	Y	N	Pt (C + SPT)	Sys
	19	39	M	Y	N	Mixed nuts	Sys

Footnote: Gender: F = female, M= male; Rhinitis: Y = Yes, N = N; Asthma: Y = Yes, N = N; Diagnosed allergy: C = suggestive clinical of allergy, OFC = positive oral food challenge that confirmed allergy, SPT = positive skin prick by prick test (>3 mm), Ig = positive specific IgE measured by inmunoCAP; Clinical: OAS = oral allergy symptoms, Sys = systemic allergy reaction, An = anaphylaxis; Nuts: Al = almond, C = cashew, Hz = hazelnut, Pi = pistachio, Pt = peanut, S = sunflower seed, Wn = walnut.

**Table 3:** Nuts included in MixNut and the patient’s specific IgE value.

	Patient Number	Nuts in the MNOFC	IgE to Pru p 3	IgE to Cor a 8	IgE to Ara h 9	Al IgE	C IgE	Hz IgE	Pi IgE	Pt IgE	S IgE	Wn IgE	Total IgE	Other Sensytizacions
**Group A**	1	Wn, Hz, Pt, S	4.7	2.6	2.7	0.5	0	2.7	0	0.5	0	0.6	44.7	No
	2	Wn, Hz, Pt, Al	1.4	0	0	0.2	0	0	0	0.1	0	0	460.0	No
	3	Wn, Hz, C, Pi,	0.4	0.2	0.2	0.1	0	0.2	0	0.2	0	0.3	100.0	No
	4	Wn, Hz, C, Pi, S	11.9	3.4	4.5	0.3	0	0.91	0	2.5	0	0.6	82.4	No
	5	Wn, Hz, C, P	0.4	0.1	0.2	2.2	0	14.5	1.30	1.1	0.9	0.6	1480	Pru p 1 1.22; Ara h 8 16.30
	6	Hz, Al	10.20	0.1	0.7	0.42	0	0.21	0.2	0.91	0.7	0.4	606.0	
**Group B**	7	C, Pi	3.7	2.2	2.8	1.6	0	3.8	0.24	3	1.9	5.7	1693.0	Pru p 4 0.25
	8	C, Pi	1.8	0.8	0.2	3.4	0	11.9	1.01	1.9	0.4	2.8	105.0	Pru p 1 10.6,
	9	C, Pi	4.3	3.2	6.1	2.4	0	2.8	0.11	1.3	1.26	5.2	113	
	10	C, Pi	8.8	1.6	2.2	1.8	0	1.78	0	1.8	0	1.6	83.9	
	11	C, Pi, Al	2.29	0	0	0.16	0	0.8	0.24	0.6	0.2	0.76	73.6	
	12	C, Pi	33.4	0.3	0.2	0.4	0	1.44	0	0.59	0.2	4.2	121	
	13	C, Pi	19.8	4.4	3.9	2.9	0.13	5.4	1.2	4.7	1.5	2.3	39.8	Pru p 4 1.03
	14	C, Pi	8.2;	3.9	2.4	2.3	0	6.9	1.3	6.3	6.1	7.6	1415	Pru p 4 5.1
	15	C, Pi, Al	2.49,	1.3	0.6	0.7	0	1.6	0.4	0.7	0	1.9	462	Cora14 0.54
	16	Pi, S	6.19	0.1	1.6	0.6	0	0	0	0.5	0	0.6	41.9	
	17	Al, Hz	10.2	0.2	0.6	0.4	0	0.2	0.2	0.9	0.7	0.4	606	Pru p 4 0.8
	18	C, Pi, Al	20.5	3.1	0.7	0.9	0	0.1	0.1	0.3	0	0.2	38.1	
	19	Nn, Pt	1.2	0.3	0.7	0	0	0.2	0	0.4	0	0.4	69.4	

Footnote: Ig = positive specific IgE measured by inmunoCAP; Nuts: Al = almond, C = cashew, Hz = hazelnut, Pi = pistachio, Pt = peanut, S = sunflower seed, Wn = walnut.

## Data Availability

The original contributions presented in this study are included in the article/Supplementary Material. Further inquiries can be directed to the corresponding author.

## Supplementary Materials

The following supporting information can be downloaded at https://www.mdpi.com/article/10.3390/nu17243822/s1. Figure S1: Specific IgE levels to LTP-related allergens and individual nuts in Groups A and B; Table S1: Specific IgE levels to LTP-related allergens and individual nuts in Groups A and B.

## Abbreviations

The following abbreviations are used in this manuscript:

AAAI	American Academy of Allergy, Asthma, and Immunology
Al	Almond
C	Cashew
EAACI	European Academy of Allergy and Clinical Immunology
Hz	Hazelnut
IgE	Immunoglobulin E
LTP	Lipid transfer protein
MixNut	Mixed nut
MNOFC	Mixed nut oral food challenge
OFC	Oral food challenge
OAS	Oral allergy syndrome
Pi	Pistachio
Pt	Peanut
S	Sunflower seed,
SD	Standard deviation
SPTs	Skin prick tests
Wn	Walnut
