# Supplementary material for: Mixed Nut Challenge Test (MixNut) as an Efficient Procedure in the Management of Lipid Transfer Protein Allergy
Source: Nutrients. 2025 Dec 6;17(24):3822. doi: 10.3390/nu17243822 (PMC12735923; doi:10.3390/nu17243822)
Supplement: Supplementary file 1 [file nutrients-17-03822-s001.zip › nutrients-4002001-supplementary.pdf]

nutrients

MDPI

Table S1: Specific IgE levels to LTP-related allergens and individual nuts in Groups A and B

Group	Allergen		Mean (kUA/L)	SD	Q1	Q3
Group A	Total IgE		462.18	548.88	86.8	569.5
	IgE to LTP allergens	Pru p 3	4.83	5.10	0.65	8.83
		Cor a 8	1.07	1.52	0.10	2.00
		Ara h 9	1.38	1.82	0.20	2.20
	IgE to individual nuts	Al	0.62	0.79	0.23	0.48
		C	0.00	0.00	0.00	0.00
		Hz	3.09	5.68	0.20	2.25
		Pi	0.25	0.52	0.00	0.15
		Pt	0.89	0.88	0.28	1.05
		S	0.27	0.42	0.00	0.53
		Wn	0.42 *	0.24	0.33	0.60
Group B	Total IgE		373.97	554.64	99.72	824.5
	IgE to LTP allergens	Pru p 3	9.45	9.58	2.16	9.15
		Cor a 8	1.64	1.55	0.20	2.35
		Ara h 9	1.69	1.77	0.20	2.35
	IgE to individual nuts	Al	1.35	1.12	0.45	2.25
		Pi	0.36	0.47	0.08	0.43
		Pt	1.78	1.85	0.83	1.82
		S	0.94	1.67	0.20	0.99
		Hz	2.84	3.46	1.28	5.82
		Wn	2.58 *	2.38	0.72	4.45

Footnote: Nuts: Al=almond, C=cashew, Hz= hazelnut, Q1= first quartile; Q3 = thirs quartile; Pi=pistachio, Pt= peanut, S=sunflower seed, SD: Standard desviation; Wn=walnut;

* means statistically difference between group A and B ($p<0.05$ is considered as a statistically significant).

Figure S1: Specific IgE levels to LTP-related allergens and individual nuts in Groups A and B

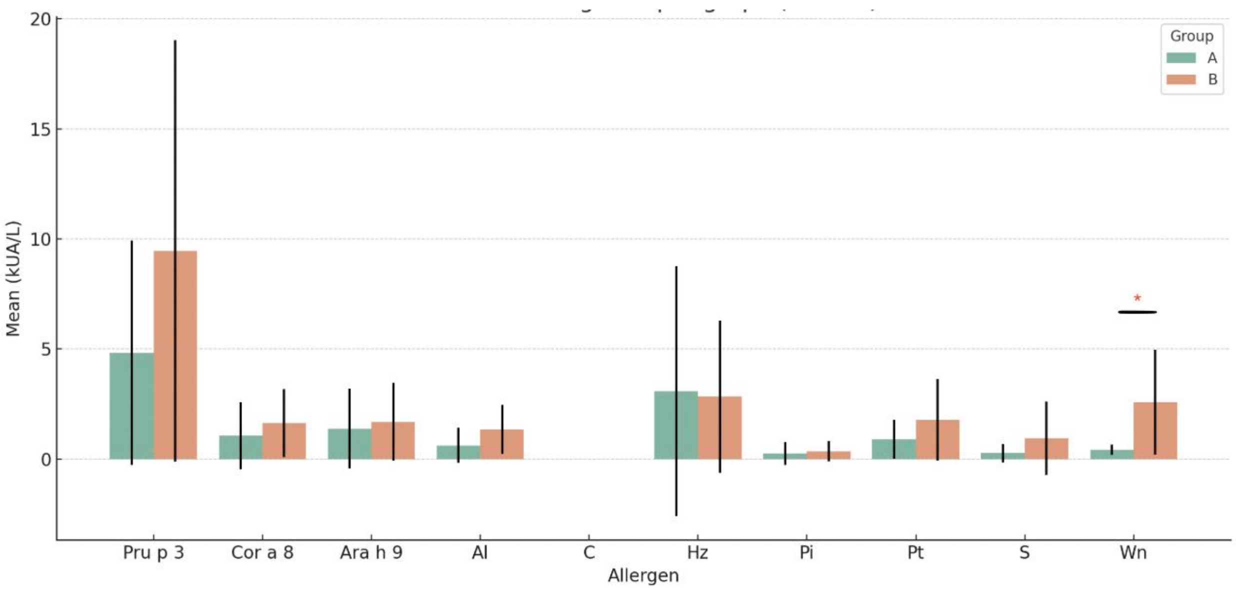

Footnote: Nuts: Al=almond, C=cashew, Hz= hazelnut, Q1= first quartile; Q3 = thirs quartile; Pi=pistachio, Pt= peanut, S=sunflower seed, SD: Standard desviation; Wn=walnut;

* means statistically difference between group A and B ($p<0.05$ is considered as a statistically significant).
